# The two kinases, AbrC1 and AbrC2, of the atypical two-component system AbrC are needed to regulate antibiotic production and differentiation in *Streptomyces coelicolor*

**DOI:** 10.3389/fmicb.2015.00450

**Published:** 2015-05-12

**Authors:** Héctor Rodríguez, Sergio Rico, Ana Yepes, Elsa Franco-Echevarría, Sergio Antoraz, Ramón I. Santamaría, Margarita Díaz

**Affiliations:** ^1^Departamento de Microbiología y Genética, Instituto de Biología Funcional y Genómica, Consejo Superior de Investigaciones Científicas, Universidad de SalamancaSalamanca, Spain; ^2^Institute for Molecular Infection Biology, Julius-Maximilians-Universität WürzburgWürzburg, Germany; ^3^Instituto de Química Física “Rocasolano”, Consejo Superior de Investigaciones CientíficasMadrid, Spain

**Keywords:** *Streptomyces*, two-component systems, antibiotic production, histidine kinases, heterologous production

## Abstract

Two-component systems (TCSs) are the most important sensing mechanisms in bacteria. In *Streptomyces*, TCSs-mediated responses to environmental stimuli are involved in the regulation of antibiotic production. This study examines the individual role of two histidine kinases (HKs), AbrC1 and AbrC2, which form part of an atypical TCS in *Streptomyces coelicolor*. qRT-PCR analysis of the expression of both kinases demonstrated that both are expressed at similar levels in NB and NMMP media. Single deletion of *abrC1* elicited a significant increase in antibiotic production, while deletion of *abrC2* did not have any clear effect. The origin of this phenotype, probably related to the differential phosphorylation ability of the two kinases, was also explored indirectly, analyzing the toxic phenotypes associated with high levels of phosphorylated RR. The higher the AbrC3 regulator phosphorylation rate, the greater the cell toxicity. For the first time, the present work shows in *Streptomyces* the combined involvement of two different HKs in the response of a regulator to environmental signals. Regarding the possible applications of this research, the fact that an *abrC1* deletion mutant overproduces three of the *S. coelicolor* antibiotics makes this strain an excellent candidate as a host for the heterologous production of secondary metabolites.

## Introduction

Two-component systems (TCSs) are the prokaryotic equivalent of human senses. In bacteria, most environmental information processing and the subsequent responses are carried out via these highly complex mechanisms. Typically, TCSs are composed of a membrane-bound histidine kinase (HK) that responds to specific signal inputs and a cognate response regulator (RR), which mediates the cellular response, mainly through transcriptional regulation of its target genes (Mascher et al., 2006).

Due to the ever-changing conditions of its natural niches, the genomes of *Streptomyces* contain a high number of TCSs so that the organism can adapt to such fluctuating situations. In the case of *S. coelicolor*, the model *Streptomyces* species, this harbors a high percentage of regulatory genes (approximately 12.3% of the total ORFs) (Bentley et al., 2002; Hutchings et al., 2004). One of the adaptive responses regulated by TCS is secondary metabolite production, as is the case with antibiotics. The role of TCSs in antibiotic production has been extensively studied and TCS manipulation offers amazing possibilities for the improvement of the discovery and production of new antibiotics (Rodríguez et al., 2013).

The involvement of AbrC1/C2/C3 TCS in the regulation of antibiotic production was initially identified in a screening for homologs to the AbsA1/2 system in the *S. coelicolor* genome (Yepes et al., 2011). This AbrC1/C2/C3 TCS system may be considered atypical owing to the presence of two different HKs in the vicinity of the regulator. Moreover, the three genes of the system are separated from each other by DNA regions, which are long enough to harbor their own promoters (114 bp between *abrC1*-*C2* and 308 bp between *abrC2-C3*). Therefore, each gene might be expressed independently in order to meet the different needs of the bacterium. Interestingly, this system is conserved in most *Streptomyces* species sequenced so far, indicating a strong likelihood that this TCS would be crucial for *Streptomyces*. Both kinases share high sequence similarity (83 and 57% nucleotide and amino acid sequence identity, respectively).

Deletion of the entire cluster (Δ*abrC1/C2/C3*) in *S. coelicolor* M145 (the parent strain) revealed that the mutant was clearly impaired in antibiotic production (actinorhodin, ACT; undecylprodiginine, RED, and calcium dependent antibiotic, CDA) and showed delayed morphological development. Therefore, the AbrC1/C2/C3 system has positive effects on both morphological development and antibiotic production (Yepes et al., 2011). To further explore the importance of the system RR (*abrC3*), a single deletion mutant of this gene (Δ*abrC3*) was also constructed and its phenotype was found to be similar, but not identical, to the one observed in the whole-system deletion mutant. ACT and RED production were strongly diminished in both the triple and single mutants as compared to the parent strain on nutrient agar medium. In contrast, CDA production in the mutant lacking only the RR, *ΔabrC3*, was unaffected, while it was clearly diminished in the triple mutant. It seems that system HKs might phosphorylate another RR, affecting CDA production. One putative candidate for phosphorylation by the HK system may be the orphan RR SCO2281, which Wang et al. have proposed to be recognized by AbrC2 (Wang et al., 2009). Microarray and ChIP-chip experiments have demonstrated that the main targets of the AbrC3 regulator correspond to *actII-ORF4*, which encodes the cluster-situated regulator (CSR) of the ACT cluster, and the sIHF gene, *afsS*, and *absR1*, which are also related to antibiotic regulation (Rico et al., 2014a).

In this study we performed an expression profile experiment of the two HK-encoding genes *abrC1* and *abrC2* in different media and at different growth times in order to decipher whether the regulation of both of them was specific. Moreover, to understand the individual role of each HK, AbrC1, and AbrC2, in antibiotic production and the morphological development of *S. coelicolor*, single-deletion mutants of each HK gene were constructed. Finally, using an indirect approach we studied the role of the two proteins in AbrC3 phosphorylation. These results provide valuable information regarding the importance of each kinase in the functioning of our TCS, also opening up new possibilities for the use of some of the strains for the heterologous expression of valuable secondary metabolites.

## Materials and methods

### Strains and growth conditions

The bacterial strains and plasmids used in this study are shown in Tables S1, S2. *E. coli* strains were grown either on solid or in liquid Luria-Bertani medium and SOB medium (Sambrook et al., 1989) at 37°C, and were supplemented with the following antibiotics: 100 μg/mL ampicillin, 50 μg/mL kanamycin, 25 μg/mL chloramphenicol, 50 μg/mL apramycin, and 25 μg/mL nalidixic acid when needed. *Streptomyces* strains were grown at 30 °C on NA liquid and agar medium (Scharlau), NMMP medium (Hopwood et al., 1985), Lechevalier medium modified from asparagine minimal medium (Martín and McDaniel, 1975), PGA medium (Coco et al., 1991), R2YE (R5) (Kieser et al., 2000), SFM (MSA), TSB (Hopwood et al., 1985), YES (Rodríguez et al., 2005), and YEPD (Rose et al., 1990), and were supplemented with 50 μg/mL kanamycin, 10 μg/mL ampicillin or 10 μg/mL thiostrepton when needed.

### Nucleic acid manipulations

Plasmid isolation, restriction enzyme digestion, ligation, and transformation of *E. coli* and *S. coelicolor* were carried out as described in Sambrook et al. (1989) and Kieser et al. (2000) respectively. The plasmids and cosmids used are listed in Table S2. Total genomic DNA from *S. coelicolor* (gDNA) was isolated from 24 to 36 h cultures in TSB medium following the procedure described in Hopwood et al., but scaled to 1–2 g of mycelium (Hopwood et al., 1985).

When needed, the DNA was sequenced on both strands using a Perkin Elmer ABI Prism 377 DNA sequencer. *In silico* plasmid designs were obtained with the Gene Construction Kit software (GCK, Texco).

### Mutant constructions

The PCR-targeted system established by Gust et al. (2003) was used to replace the complete coding sequences of *abrC1* (*SCO4598*) and/or *abrC2* (*SCO4597*) by the apramycin resistant gene *aac(3)IV*, using cosmid SCD20 (http://streptomyces.org.uk/). The primers used to amplify the mutagenic cassette using pIJ773 as template are listed in Table S3. The mutated cosmids SCD20*ΔabrC1::acc(3)IV* (*ΔSCD20-3*), SCD20*ΔabrC2::acc(3)IV* (*ΔSCD20-4*) and SCD20*ΔabrC1/C2::acc(3)IV* (*ΔSCD20-5*) obtained in *E. coli* BW25113(pIJ790) were demethylated in the ET12567(pUZ8002) strain and transferred by conjugation to *S. coelicolor* M145. The desired double recombinants carrying apramycin resistance, while being sensitive to kanamycin (the selection marker for the vector sequences), were selected. Southern blotting and PCR assays confirmed the deletion of the *abrC1* and/or *abrC2* genes in the *S. coelicolor* M145 mutant strains obtained.

### Plasmid constructions

The integrative plasmid pSETabrC1 was constructed by cloning the *abrC1* gene under the regulation of its own promoter into the *Streptomyces* integrative shuttle plasmid pSET152t using NdeI and BamHI restriction sites. This plasmid was used for the complementation of the *ΔabrC1* mutant strain (Table S2).

Multicopy plasmids -pNXabrC3, pNXabrC3-DA, pNXabrC3-DE, and pNXabrC3-DADE- were derivatives of the pN702GEM3 plasmid (Fernández-Abalos et al., 2003). In this shuttled (*E. coli-Streptomyces*) multicopy plasmid, the different mutant versions of the *abrC3* gene were controlled by the xylanase promoter *xysAp* (Rodríguez et al., 2005). To obtain pNXabrC3, the gene was amplified using SRG-001 (introducing an NdeI site) and SRG-002 (introducing an XhoI site) oligonucleotides (see Table S3) and the amplicon was introduced into the same sites of pXHis1 (Adham et al., 2001), obtaining an intermediate plasmid, pXabrC3His. An NdeI/HindIII fragment from this plasmid was then cloned into the same sites of pNX24 (pN702GEM3 derivative). To obtain plasmids pNXabrC3-DA and pNXabrC3-DE, point mutations D_61_A or D_61_E were introduced using a pair of oligonucleotides specific for each mutation in the first step of an overlapping PCR: SRG-001/AY-094 (5′ gene fragment DA) and AY-093/SRG002 (3′ gene fragment DA) or SRG-001/SRG055 (5′ gene fragment DE) and SRG-054/ SRG-002 (3′ gene fragment DE). Following this, using these two overlapping fragments for D_61_A or D_61_E as templates, a second PCR with SRG001/SRG002 was carried out, and the final mutated genes were introduced by NdeI/XhoI digestion, replacing the non-modified version carried in pNXabrC3. In pNXabrC3-DADE, the mutant gene (D_12_A/D_61_E) was amplified using SRG-061/SRG-002 oligonucleotides and pNXabrC3-DE plasmid as the template. This double mutant gene fragment was substituted as above in pNXabrC3.

The new plasmids were introduced into the corresponding *Streptomyces* strains by protoplast transformation, as previously described (Kieser et al., 2000). Plasmids pET_c_abrC1 and pET_c_abrC2, containing the cytoplasmic regions of AbrC1 (cAbrC1:180–407 aminoacids, 25,9 kDa) and AbrC2 (cAbrC2: 227–455 aminoacids, 24,9 kDa), were obtained using PCR amplification with oligonucleotides AY-097/AY-098 and AY-095/AY-096 and were then cloned into the NdeI/XhoI sites of the polylinker of pET22(b).

Point mutations were introduced in the cytoplasmatic regions of both HKs (*_c_abrC1* and *_c_abrC2*) inserted into plasmids pET_c_abrC1 and pET_c_abrC2 to replace H214 and H270 respectively for an Ala, obtaining plasmids pET_c_abrC1_H_ and pET_c_abrC2_H_. The mutations were performed with PCR, using overlapping primers containing the mutation (SRG-005/SRG-006). PCR was then digested with DpnI and the digestion product was transformed into DH5α *E. coli*. In order to confirm that the mutations were correct, different colonies were selected and their plasmids were isolated and sequenced.

### HK autophosphorylation

Autophosphorylation assays were carried out with 10 μCi of **γ**^32^ATP (6000 Ci/mmol Perkin Elmer, Wellsley, MA) in phosphorylation buffer (Tris 50 mM, pH 7,5, NaCl 100 mM, MgCl_2_ 10 mM) in a final volume of 20 μL. After the addition of 2 μg of protein, samples were incubated for 15 and 30 min at 30°C. Phosphorylation reactions were stopped by the addition of SDS-PAGE buffer without DTT. Samples were then loaded on 15% polyacrylamide and gel electrophoresis was performed. In order to visualize the phosphorylated bands properly, the gels were covered with transparent film and developed with autoradiography using either a Kodak photographic film or a BAS-IPMP (Fujifilm) screen acquired with a BAS-1500 phosphorimager.

### Antibiotic determination

Antibiotic production was assayed on solid media as described by Uguru et al. (2005). Plates were inoculated with 5 × 10^5^ spores in a 5-μL drop. For CDA production, the strains were grown on NA medium at 30°C for 2 days. Then, the plates were overlaid with 5 mL of soft agar plus 60 mM Ca(NO_3_)_2_ inoculated with *B*. *subtilis* as the test microorganism (0.2 mL, 0.25 DO_600_) and were incubated at 30°C for 20 h. A replica plate without calcium was used as a negative control. For ACT production on solid media, the strains were grown on different media at 30°C for at least 3 days to observe the blue halo around the colonies. RED production was detected as the red color of the colonies on different media after 2 days.

ACT and RED antibiotic production was quantified in liquid cultures using the standard spectrophotometric method (Kieser et al., 2000) with minor modifications. 30 mL of medium were inoculated with 5 × 10^5^ spores/mL. Culture samples were mixed with the same volume of 1N KOH and incubated overnight at 4°C, centrifuged (15,000 g, 10 min), and the absorbance at 640 nm of the supernatants were determined to quantify ACT (ε_640_ = 25,320). To quantify RED, pellets were washed twice in 0.5 M HCl and extracted in 0.5 M HCl-methanol for 2 h. After centrifugation (15,000 g, 5 min), supernatant absorbance at 530 nm was measured (ε_530_ = 100,500). The dry weight of samples was measured at different times to monitor culture growth.

### RNA isolation

For RNA extraction from *S. coelicolor* M145, 160 mL of NB, and NMMP media was inoculated in 500 mL three-baffle flasks with 4 × 10^6^ spores/mL and incubated at 30°C for 36 and 48 h in NB medium and for 48 and 72 h in NMMP medium. Two biological replicas and three technical ones were used for each time-point. Prior to RNA isolation, 20 mL of culture was harvested and suspended in RNA-protect Bacteria Reagent (Qiagen). Following mycelium lysis with lysozyme (15 mg/mL in TE at room temperature), three volumes of RLT buffer from the RNeasy mini plus kit (Qiagen) were added, and mycelia were disrupted using Fast-prep (2 pulses maximum intensity, and introduction in ice for 5 min between cycles). The lysate was clarified using centrifugation, and the RNeasy mini Plus (Qiagen) kit was used to purify the RNA following the manufacturer's specifications. The quality and concentration of RNA were assayed using spectrophotometric assays (Nanodrop ND1000).

### qRT-PCR

Specific primers and probes for the genes tested were designed using the Primer3 web-based tool (Table S3). Five-μg RNA samples were treated with RNAase-free DNaseI (Promega) according to the manufacturer's instructions. One μg of the resulting RNA was used as template for cDNA synthesis, using iScript Reverse Transcription Supermix for RT-qPCR (Bio-Rad) in 20 μL reaction volumes. The samples were diluted 1:1 with distilled water and 5 μL was used in the quantitative PCR reaction with 10 pmol of forward and reverse primer, 2.5 pmol of FAM/TAMRA dual-labeled specific probe, and 12.5 μL SsoFastTM Probes Supermix with ROX master mix (Bio-Rad) in a final volume of 25 μL. For the operon expression studies, SsoAdvanced SYBR Green Supermix (Bio-rad) was used instead of probes. In the PCR reaction, 10 uL of the mix was added to 10 pmol of forward and reverse primer using cDNA in the same concentration as in the probe assays.

Each assay was performed in duplicate using the CFX96 Touch™ Real-Time PCR Detection System (Bio-Rad). Control PCRs were included to check that there was no DNA contamination (without RT, enzyme negative control). Relative quantification of gene expression was performed with the ΔΔCt method (Livak and Schmittgen, 2001; Rico et al., 2014a). The expression levels of the target genes were normalized using the *hrdB* gene (*SCO5820*) as an internal control to quantify the relative expression of the target genes, since its expression level remained constant under all the conditions analyzed in a previous microarray performed by the group. Absolute quantification was performed using decimal serial dilutions of *S. coelicolor* genomic DNA with a known number of copies as standard.

## Results

### Expression profiles of the *abrC1* and *abrC2* HKs

In order to determine whether the genes of the AbrC TCS were expressed simultaneously or not, the individual expression of each gene was analyzed in two different media and at two different culture time-points. NB and NMMP media were used to grow the parental strain, *S. coelicolor* M145, since the phenotypes of the triple mutant (Δ*abrC1/C2/C3*) and the single RR mutant (Δ*abrC3*) had previously been observed to be medium-dependent (Rico et al., 2014a). Culture samples were taken at 36/48 or at 48/72 h, depending on the growth rates in each single medium (NB/NMMP respectively) and qRT-PCR was performed (see Materials and Methods).

**Figure 1:**
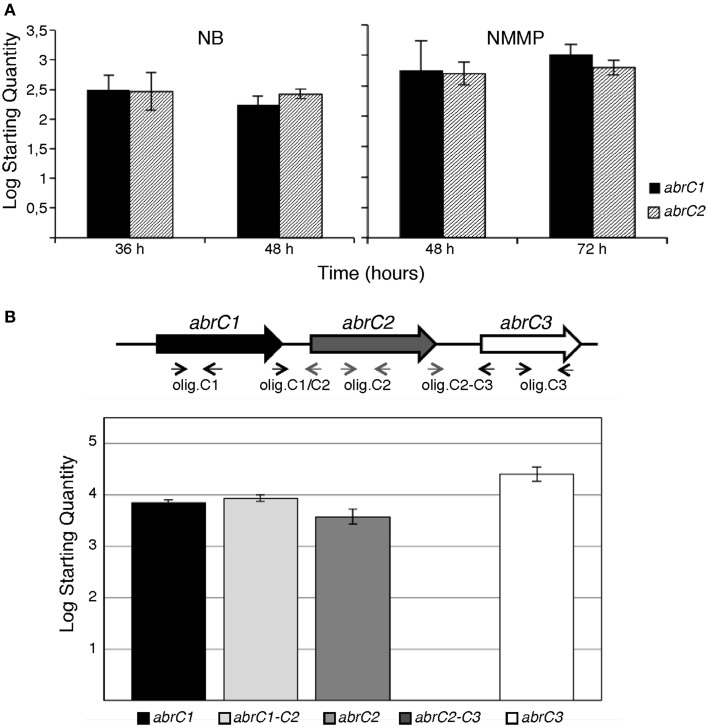
**q-RT-PCR of AbrC genes in *S. coelicolor* M145. (A)** Absolute quantification of *abrC1* and *abrC2* expression: qRT-PCR expression profile of both HK-encoding genes in NB and NMMP media at two different culture times. **(B)** q-RT-PCR of the putative AbrC operon genes. Top: scheme of the gene structure of the putative AbrC operon and location of the oligonucleotides used. Bottom: expression profile found by qRT-PCR of the three genes of the putative AbrC operon and the corresponding intergenic regions between the different genes in NB medium at 48 h. Data represent the Log Starting Quantity of each sample.

Absolute quantification was applied to study the differential expression of both HK-encoding genes in the same sample and at the same time-point. The qRT-PCR results revealed that both genes were expressed under all conditions tested. Moreover, the expression levels of *abrC1* and *abrC2* were similar in both media and growth times tested (Figure 1A).

Similarly, relative quantification was used to measure the differential expression of both genes under different temporal and medium conditions. In all cases, the *hrdB* housekeeping gene was used as a reference. Regarding the culture growth time-point, the expression of both genes in both media (NB and NMMP) was slightly higher at longer growth periods (48 h for NB and 72 h for NMMP) (Table 1). Measurements of expression comparing these different media were also performed. NB complex medium afforded comparatively higher expression values than NMMP medium at all time-points checked (Table 1).

**Table 1:** **Relative quantification of the differential expression of *abrC1* and *abrC2* by qRT-PCR in different culture time-points and media**.

**Gene**	**Differential expression (fold-change)**			
	**Culture media**		**Time**	
	**First time samples (NB-36 h vs. NMMP-48 h)**	**Second time samples (NB-48 h vs. NMMP-72 h)**	**NB (48 h vs. 36 h)**	**NMMP (72 vs. 48 h)**
*abrC1*	2.32	1.91	1.91	1.26
*abrC2*	6.6	3.6	1.14	1.94

These similar expression levels of *abrC1* and *abrC2* observed under in all conditions tested suggested a common regulation for both genes. Although these genes are separated by 114 bp, sufficient space to locate a putative independent promoter, there also existed the possibility that both genes might be transcribed together, constituting a single operon. In order to test this, the presence of a putative transcript containing the *abrC1* and *abrC2* genes was studied via qRT-PCR. Intergenic region amplification was observed under all conditions assayed, confirming that both genes were transcribed together in a single transcriptional unit. However, no transcript was observed for the region between the AbrC2 kinase and the AbrC3 regulator, indicating that the regulator was expressed independently (Figure 1B).

### Phenotypic characterization of the Δ*abrC1* and Δ*abrC2* mutants

**Figure 2:**
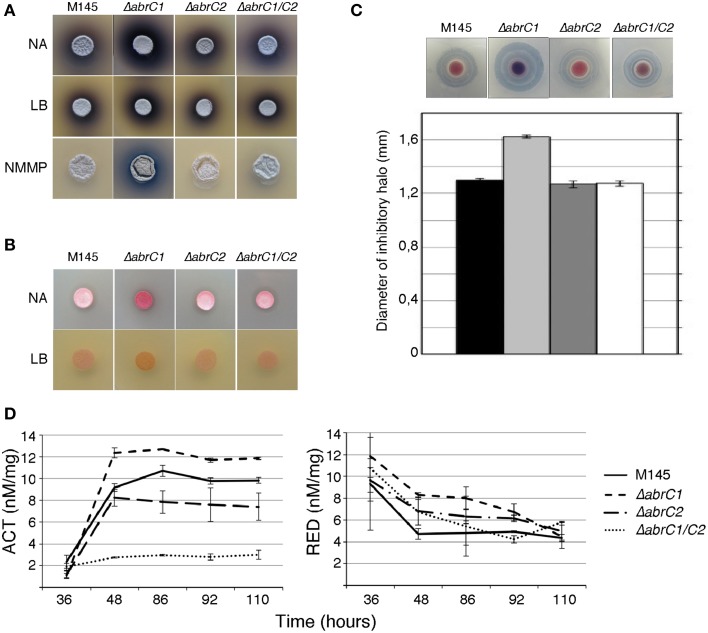
**Antibiotic production and morphological differentiation of the AbrC1 and AbrC2 HK mutants. (A)** Production of ACT at 4 days of growth on NA, LB, and NMMP. **(B)** Production of RED at 2 days of growth on NA and LB. **(C)** Calcium-dependent antibiotic (CDA) production in a bioassay against *B. subtilis* at 2 days of growth and its inhibition halo diameter in the lower part. **(D)** Quantification of ACT and RED production in liquid NB medium. Error bars correspond to four different replicates.

With a view to deciphering the specific role of each individual HK in the positive antibiotic and morphological regulation cascade triggered by the AbrC TCS, the *S. coelicolor* M145 deletion mutants Δ*abrC1* and Δ*abrC2* and the double deletion mutant Δ*abrC1/C2* were generated according to the PCR-targeting approach described by Gust et al. (2003) (see Materials and Methods). Deletions were checked by Southern blot and PCR. Since the activating signal of the system remained obscure, different solid media were used to study possible phenotypic differences between the mutants and the parent strain M145 regarding antibiotic production and morphological differentiation. Thus, RED, ACT and CDA production was studied on solid media at different culture time-points. Of all solid media tested (see Materials and Methods), differential phenotypes were observed in only three: NA, LB and NMMP (Figure 2A). The Δ*abrC1* deletion mutant showed a significant hyper-production of ACT in NB, NMMP and LB media with respect to the parent strain, while the deletion mutant Δ*abrC2* and the double mutant Δ*abrC1/C2* showed parent strain levels. Similar hyper-production patterns were observed in *ΔabrC1* for RED production in NA and LB media (Figure 2B) and for CDA in NA medium (Figure 2C).

In order to confirm the results obtained with solid media, antibiotic quantification was also performed in NB liquid media (see Materials and Methods for the quantification protocol). Both ACT and RED overproduction in Δ*abrC1* was also evident in liquid media. Regarding Δ*abrC2*, the RED production of the mutant was slightly higher than that of the parent strain in liquid media, while by contrast ACT production was slightly impaired in comparison with the parent strain (Figure 2D). The double HK mutant showed parent-strain production of RED but, and surprisingly, a marked decrease in ACT production. Here we only show the NB liquid media results because the phenotypes were similar in all three media.

All the mutant strains showed growth rates similar to that of the wild-type M145 strain in NB medium, indicating that the different degree of antibiotic production cannot be attributed to growth impairments (Figure S1). Polar effects were also discarded as the origin of antibiotic overproduction in Δ*abrC1*. The complementation of the Δ*abrC1* strain with the pSETabrC1 plasmid recovered the antibiotic production levels of the M145.pSET152t strain (pSET152t: empty plasmid used as control) under all conditions tested (Figure S2).

### HK autophosphorylation activity

**Figure 3:**
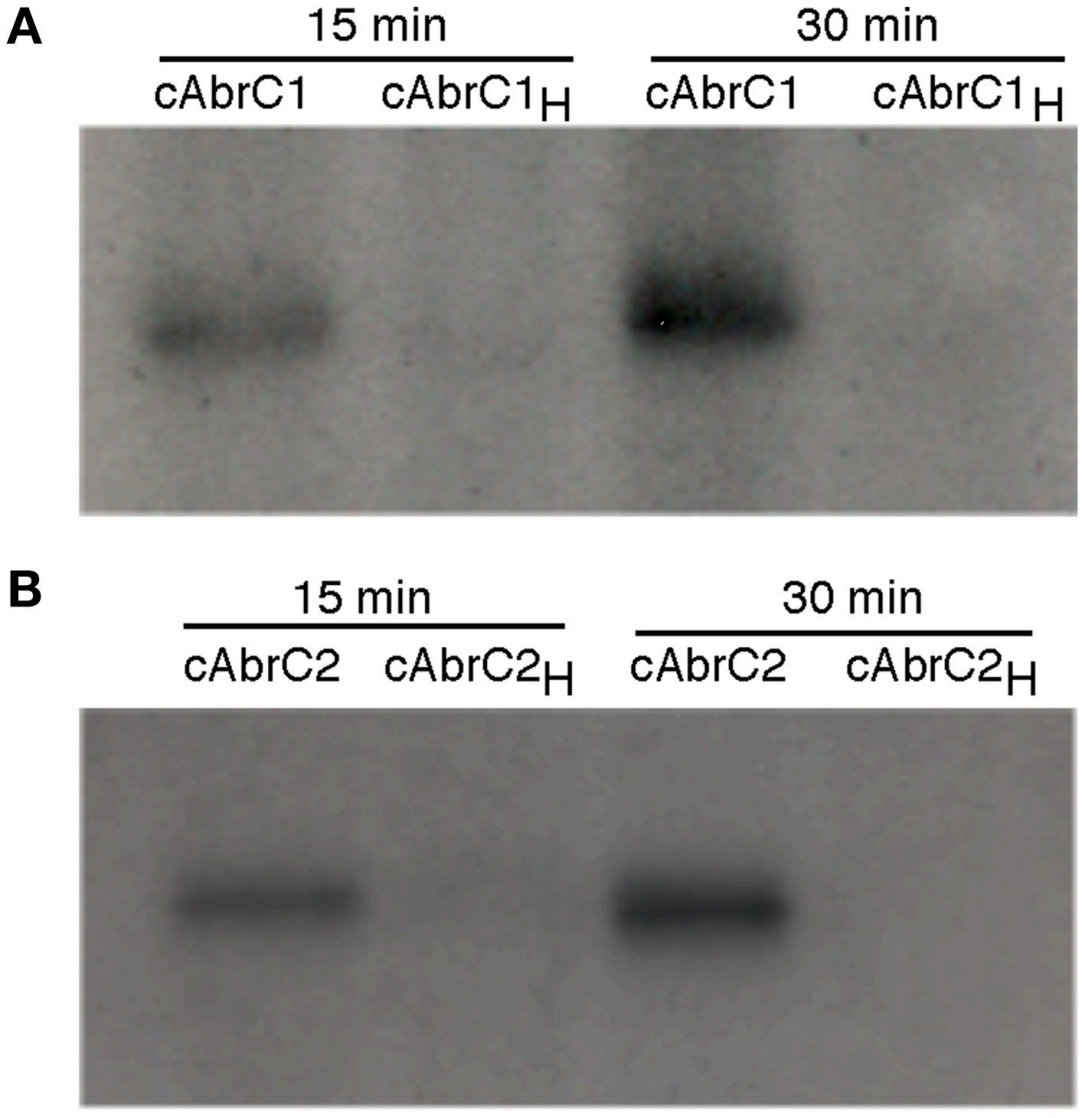
**Autophosphorylation of AbrC1 and AbrC2 kinases**. Auto-phosphorylation assays of AbrC1 **(A)**, and AbrC2 **(B)** with **γ**^32^-P after 15 and 30 min of reaction. The proteins used were the cytoplasmic domains of both proteins (_c_AbrC1 and _c_AbrC2) and their variants with the corresponding phosphorylizable histidine (H_214_ in AbrC1 and H_270_ in AbrC2) replaced by an alanine (_c_AbrC1_H_ and _c_AbrC2_H_).

In order to confirm that both HKs were functional, auto-phosphorylation assays were carried out. DNA corresponding to the cytoplasmic domains of both HKs (cAbrC1 and cAbrC2) with a Hisx6-tag were cloned into *E. coli* BL21(DE3), and the purified proteins were incubated with radioactive phosphorus labeled ATP (**γ**-^32^P) as described in Materials and Methods. Samples were resolved on polyacrylamide gels and developed. The autoradiography confirmed that selected cytoplasmic fractions of each HK maintained their phosphorylation capacity. Furthermore, an increase in the incubation time resulted in a higher HK phosphorylation level (Figure 3). In order to accurately define the His-residue responsible for the previously observed auto-phosphorylation, an alignment of the two HKs with the AbsA1 HK, in which the histidine phosphorylizable residue had been confirmed experimentally, was performed (Sheeler et al., 2005). The corresponding putative histidine of each HK (H_214_ in AbrC1 and H_270_ in AbrC2) was defined and replaced by directed mutagenesis by a non-phosphorylizable alanine residue (see Materials and Methods). The phosphorylation reactions performed with the resulting purified protein variants cAbrC1_H_ and cAbrC2_H_ (Figure 3) showed that proteins lacking the canonical histidines could not be phosphorylated, confirming the above residues as the phosphorylizable ones.

### HK activity role in the AbrC TCS

The *in vitro* ability of both HKs to transfer phosphate to the RR AbrC3 did not afford clear results. These inconsistent results were probably originated by the aggregates formed by overproduced AbrC3. Accordingly, as an alternative we used an *in vivo* indirect approach.

**Figure 4:**
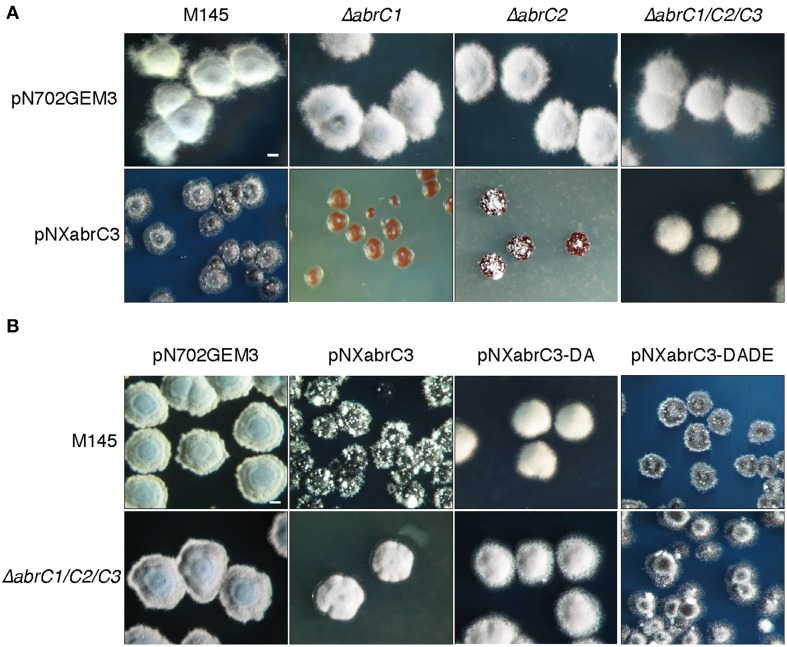
**Overexpression of *abrC3*. (A)** Colony morphology of the *S. coelicolor* M145, Δ*abrC1*, Δ*abrC2*, and Δ*abrC1/C2/C3* strains transformed with the multicopy plasmids pN702GEM3 (control) and pNXabrC3 (expressing the AbrC3 RR under the control of *xysAp*). **(B)** Colony morphology of *S. coelicolor* M145 and Δ*abrC1/C2/C3* strains transformed with different multicopy plasmids: pN702GEM3, pNXabrC3, and its derivatives pNXabrC3-DA (D_61_A) and pTXabrC3-DADE (D_12_A, D_61_E). The photographs correspond to 4-day cultures on R2(YE) medium. Bar: 0.1 cm.

We observed an aberrant phenotype of a *S. coelicolor* M145 strain overexpressing the *abrC3* gene with plasmid pNXabrC3 (Figure 4A). These morphologically aberrant colonies grew slower than the M145 strain and it was not possible to subculture them by re-inoculating them onto fresh agar plates or into liquid medium. Taking advantage of this toxic phenotype as a measure of the functional AbrC3 RR, and in order to decipher the role of the cognate HKs in the RR, we also overexpressed *abrC3* in the Δ*abrC1*, Δ*abrC2*, and Δ*abrC1/C2/C3* backgrounds (Figure 4A).

The growth of Δ*abrC1/C2/C3* (pNXabrC3) revealed colonies with a remarkably normal appearance, although slightly smaller than the control strain (only with the pN702GEM3 vector), indicating that the presence of both kinases that phosphorylate AbrC3 was necessary for the toxic phenotype to be observed. Besides, the overexpression of *abrC3* in the single mutants Δ*abrC1* and Δ*abrC2* was even more toxic than in the M145 strain and, remarkably, RR overexpression in Δ*abrC1* was much more toxic than in the Δ*abrC2* deletion mutant, indicating that the two kinases had different capacities to phosphorylate the RR (Figure 4A).

To further explore this, the overexpression was repeated with a phosphoablative protein version of AbrC3 in which, the putative site of phosphorylation, residue Asp-61 was replaced by an Ala (plasmid pNXabrC3-DA: D_61_A). The results showed no toxicity, either in the parent strain or in the triple mutant (Figure 4B). Additionally, we generated a phosphomimetic version of AbrC3, which was always active, independently of phosphorylation. To accomplish this, we replaced the Asp-12 of the acidic pocket with Ala and residue Asp-61 with Glu (AbrC3-DADE: D_12_A/D_61_E), following comparable work carried out with *E. coli* PhoB (Arribas-Bosacoma et al., 2007). As shown in Figure 4B, overexpression of the AbrC3-DADE encoding gene was toxic in both strains (M145 and triple mutant) and hence this protein variant was considered to be always active, even in the absence of its phosphorylating HKs, AbrC1, and/or AbrC2.

## Discussion

In this paper, we have attempted to advance our knowledge of the role of the two independent HKs (AbrC1 and AbrC2) present in the AbrC TCS. The presence of two HKs in the same system is unusual in TCSs. In spite of the DNA regions located upstream of each gene, which could act as putative independent promoter regions, we failed to observe the differential expression of either HK in any of the media or conditions tested. This was shown via qRT-PCR experiments quantifying their expression profiles. The similar expression levels of *abrC1* and *abrC2* in all conditions tested pointed to a common regulation for both genes and their possible co-expression from a single promoter. The co-expression of both kinase genes was demonstrated by qRT-PCR, while no transcript was observed for the intergenic region between the AbrC2 kinase and the AbrC3 regulator, indicating that the regulator is transcribed independently. Interestingly, in a recent publication we have shown that the AbrC3 regulator exhibits auto-regulation on its own promoter (Rico et al., 2014a) but it does not produce any effect on the expression of either HK. The fact that both kinase genes were co-transcribed suggests that both proteins are always crucial for external stimuli to be received and processed and for the correct level of phosphorylated AbrC3 to be maintained. Taking the qRT-PCR results into consideration, it might also be inferred that HKs expression would be constitutive at different time-points and in different media. Constitutive expression of these proteins indicates that their functional regulation probably takes place predominantly at post-translational level.

The single deletion mutant of each kinase revealed the different, and perhaps complementary, role of both. In this sense, Δ*abrC1* showed a clear increase in ACT, RED and CDA antibiotic production while Δ*abrC2* exhibited the parental strain levels of production for all three antibiotics. Considering that the AbrC TCS acts as an antibiotic activator through its AbrC3 RR, the antibiotic overproduction observed in Δ*abrC1* suggests a more important role in the phosphorylation of AbrC3 by the other kinase, AbrC2, than by AbrC1, as explained below. Moreover, the absence of AbrC1 elicits an antibiotic hyper-production, suggesting that the role of this AbrC1 HK might be to maintain the right amount of phosphorylated AbrC3, probably through phosphatase activity when both HKs are present.

Although *in vitro* HK auto-phosphorylation was observed, no clear phosphotransfer to AbrC3 was seen. However, the phosphorylation capacity of both kinases can be inferred from the effect of AbrC3 overexpression in the different strains. Thus, overexpression in the wild-type strain or in the strains lacking one of the kinases produces a toxic effect, while overexpression in the triple mutant, lacking both kinases, does not lead to any phenotypic alterations (Figure 4). These results suggest that, although overexpressed, AbrC3 would not be active and, therefore non-toxic, when both cognate HKs are absent. Consistent with the hypothesis that AbrC2 is more efficient at phosphorylating AbrC3, the most aberrant phenotype would be expected in the most efficient phosphorylation scenario. Thus, almost total growth impairment and an absence of differentiation was observed in the Δ*abrC1* strain over-producing AbrC3. Regarding Δ*abrC2*, this also showed an aberrant phenotype, but more similar to the parental strain. It may be concluded that both HKs are functional and able to activate the regulator via phosphorylation in the absence of the other.

Our hypothesis that the lack of toxicity would be due to a non-phosphorylated state of the RR was corroborated by the observation that a phosphoablative (AbrC3-DA) mutant of the regulator did not cause any aberrant phenotype while, conversely, a phosphomimetic RR mutant (AbrC3-DADE) was always toxic when transformed in any strain, indicating that the toxicity was indeed caused by a phosphorylated, and therefore active, over-produced AbrC3.

Regarding future applications of this research, the hyper-producer strain (Δ*abrC1*) might be a good candidate as a host for heterologous antibiotic production. Similar experiments addressing the improvement of antibiotic production have been performed successfully in our lab with other strains (Rico et al., 2014b). Furthermore, we have previously shown that overexpression of the AbrC3 regulator offers the possibility of using this low-copy number plasmid to enhance antibiotic production. Another possible approach aimed at discovering cryptic antibiotic pathways might be the transformation of different *Streptomyces* strains with a plasmid overexpressing the AbrC3 regulator. As has been shown previously, HK overexpression can be used to unveil cryptic antibiotic biosynthesis (McKenzie et al., 2010).

## Author contributions

Conceived and designed the experiment: HR, SR, EF, AY, SA, RS, and MD. Performed the experiments: HR, SR, EF, SA, and AY. Analyzed the data: HR, SR, EF, AY, SA, RS, and MD. Wrote the paper: HR, RS, and MD. Revised and approved the final manuscript: HR, SR, EF, AY, SA, RS, and MD.

### Conflict of interest statement

The authors declare that the research was conducted in the absence of any commercial or financial relationships that could be construed as a potential conflict of interest.
