# Supplementary material for: The two kinases, AbrC1 and AbrC2, of the atypical two-component system AbrC are needed to regulate antibiotic production and differentiation in Streptomyces coelicolor
Source: Front Microbiol. 2015 May 12;6:450. doi: 10.3389/fmicb.2015.00450 (PMC4428217; doi:10.3389/fmicb.2015.00450)
Supplement: Supplementary file 1 [file Table1.DOCX]

***Table S1: Strains used in this work***

| **Strain** | **Genotype** | **Reference** |
| --- | --- | --- |
| *S. coelicolor* A3(2) M145 | SCP1- SCP2- | ([Kieser *et al.*, 2000](#_ENREF_7)) |
| *S. coelicolor ∆abrcC1* | *S. coelicolor* M145’ derivative | This work |
| *S. coelicolor ∆abrC2* | *S. coelicolor* M145’ derivative | This work |
| *S. coelicolor ∆abrcC1/C2* | *S. coelicolor* M145’ derivative | This work |
| *S. coelicolor ∆abrcC1/C2/C3* | *S. coelicolor* M145’ derivative | ([Yepes *et al.*, 2011](#_ENREF_12)) |
| *E.coli* BW25113 | *E. coli* K 12 derivative (Δ*ara*BAD. Δ*rha*BAD) | ([Datsenko and Wanner, 2000](#_ENREF_3)) |
| *E.coli* ET12567 | *Dam, dcm, hsdS, cat, tet* | ([MacNeil *et al.*, 1992](#_ENREF_8)) |
| *E. coli* DH5α | F^-^, 80d lacZΔM15, *Δ(lacZYAargF)U169, recA1, endA1, hsdR17(rk-,mk+), supE44, λ-, thi-1, gyrA, relA1* | ([Hanahan, 1983](#_ENREF_6)) |
| *Bacillus subtilis* | Wild type strain | CECT 4522 |

**References**

Kieser, T., Hopwood, D.A., Bibb, J.M., Chater, K.F., and Buttner, M.J. (2000). *Practical Streptomyces genetics.* Norwich, UK: John Innes Foundation.

Yepes, A., Rico, S., Rodríguez-García, A., Santamaría, R.I., and Díaz, M. (2011). Novel two-component systems implied in antibiotic production in *Streptomyces coelicolor*. *PLOS ONE* 6**,** e19980

Datsenko, K.A., and Wanner, B.L. (2000). One-step inactivation of chromosomal genes in *Escherichia coli* K-12 using PCR products. *Proc Natl Acad Sci U S A* 97**,** 6640-6645.

Macneil, D.J., Gewain, K.M., Ruby, C.L., Dezeny, G., Gibbons, P.H., and Macneil, T. (1992). Analysis of *Streptomyces avermitilis* genes required for avermectin biosynthesis utilizing a novel integration vector. *Gene* 111**,** 61-68.

Hanahan, D. (1983). Studies on transformation of *Escherichia coli* with plasmids. *Journal of Molecular Biology* 166**,** 557-580.
