# Supplementary material for: The two kinases, AbrC1 and AbrC2, of the atypical two-component system AbrC are needed to regulate antibiotic production and differentiation in Streptomyces coelicolor
Source: Front Microbiol. 2015 May 12;6:450. doi: 10.3389/fmicb.2015.00450 (PMC4428217; doi:10.3389/fmicb.2015.00450)
Supplement: Supplementary file 2 [file Table2.DOCX]

***Table S2: Plasmids and cosmids used in this work***

| **Vector** | **Characteristics** | **Reference** |
| --- | --- | --- |
| pIJ790 | λ-RED (*gam, beta, exo*), *cat, araC, rep101^ts^* | ([Gust *et al.*, 2003](#_ENREF_5)) |
| pIJ773 | *aac(3)IV* (Apra^R^)+*oriT*, FRT sites | ([Gust *et al.*, 2003](#_ENREF_5)) |
| pUZ8002 | *tra, neo*, RP4 | ([Paget *et al.*, 1999](#_ENREF_9)) |
| SCD20 | Supercos-1 derivative: *bla, neo*. Contains genes *SCO4596/97/98* | ([Redenbach *et al.*, 1996](#_ENREF_10)) |
| ∆SCD20-3 | SCD20 *ΔSCOabrC1::aac(3)IV* | This work |
| ∆SCD20-4 | SCD20 *ΔSCOabrC2::aac(3)IV* | This work |
| ∆SCD20-5 | SCD20 *ΔSCOabrC1/C2::aac(3)IV* | This work |
| pXHis1 | *E. coli* plasmid Amp resistance | ([Adham *et al.*, 2001b](#_ENREF_2)) |
| pN702GEM3 | *E. coli–Streptomyces* shuttle vector;  Neo/Kan resistance | ([Fernández-Abalos *et al.*, 2003](#_ENREF_4)) |
| pNX24 | pN702GEM3 derivative. *xysA* promoter from  *S. halstedii* controls xylanase expression. | ([Adham *et al.*, 2001a](#_ENREF_1)) |
| pNXabrC3 | pN702GEM3 derivative containing *abrC3* under the control of *xysA* promoter | ([Rico *et al.*, 2014a](#_ENREF_11)) |
| pNXabrC3-DA | Similar to pNXabrC3 in which AbrC3 presents a replacement of the Asp-61 by an Ala . | This work |
| pNXabrC3-DE | Similar to pNXabrC3 in which AbrC3 presents a replacement of the Asp-61 by a Glu . | This work |
| pNXabrC3-DADE | Similar to pNXabrC3 in which AbrC3 presents a replacement of the Asp-12 by an Ala and Asp-61 by Glu . | This work |
| pSET152t | Integrative plasmid with hygromycin/tiostreptone resistance | ([Rico *et al.*, 2014a](#_ENREF_11)) |
| pSETabrC1 | pSET1 derivative derivative containing *abrC1* | This work |
| pET22b | *E. coli* expression vector. Ampicillin  resistance. | Novagen |
| pET_c_abrC1 | pET22b derivative containing a fragment of *abrC1* encoding the cytoplasmic region with a His6 tag at the carboxy terminal. | This work |
| pET_c_abrC1_H_ | pET_c_abrC1 derivative with a punctual mutation replacing histidine 214 by alanine | This work |
| pET_c_abrC2 | pET22b derivative containing a fragment of *abrC2* encoding the cytoplasmic region with a His6 tag at the carboxy terminal. | This work |
| pET_c_abrC2_H_ | pET_c_abrC2 derivative with a punctual mutation replacing histidine 270 by alanine | This work |

**References**

Adham, S.A., Campelo, A.B., Ramos, A., and Gil, J.A. (2001a). Construction of a xylanase-producing strain of *Brevibacterium lactofermentum* by stable integration of an engineered *xysA* gene from *Streptomyces halstedii* JM8. *Appl Environ Microbiol* 67**,** 5425-5430.

Adham, S.A., Honrubia, P., Díaz, M., Fernández-Ábalos, J.M., Santamaría, R.I., and Gil, J.A. (2001b). Expression of the genes coding for the xylanase Xys1 and the cellulase Cel1 from the straw-decomposing *Streptomyces halstedii* JM8 cloned into the amino-acid producer *Brevibacterium lactofermentum* ATCC13869. *Arch Microbiol* 177**,** 91-97.

Fernández-Abalos, J.M., Reviejo, V., Díaz, M., Rodríguez, S., Leal, F., and Santamaría, R.I. (2003). Posttranslational processing of the xylanase Xys1L from *Streptomyces halstedii* JM8 is carried out by secreted serine proteases. *Microbiology* 149**,** 1623-1632.

Gust, B., Challis, G.L., Fowler, K., Kieser, T., and Chater, K.F. (2003). PCR-targeted *Streptomyces* gene replacement identifies a protein domain needed for biosynthesis of the sesquiterpene soil odor geosmin. *Proc Natl Acad Sci U S A* 100**,** 1541-1546.

Paget, M.S., Leibovitz, E., and Buttner, M.J. (1999). A putative two-component signal transduction system regulates *sigmaE*, a sigma factor required for normal cell wall integrity in *Streptomyces coelicolor A3*(2). *Mol Microbiol* 33**,** 97-107.

Redenbach, M., Kieser, H.M., Denapaite, D., Eichner, A., Cullum, J., Kinashi, H., and Hopwood, D.A. (1996). A set of ordered cosmids and a detailed genetic and physical map for the 8 Mb *Streptomyces coelicolor* A3(2) chromosome. *Molecular Microbiology* 21**,** 77-96.

Rico, S., Santamaría, R.I., Yepes, A., Rodríguez, H., Laing, E., Bucca, G., Smith, C.P., and Díaz, M. (2014a). Deciphering the Regulon of *Streptomyces coelicolor* AbrC3, a Positive Response Regulator of Antibiotic Production. *Applied and Environmental Microbiology* 80**,** 2417-2428.
