# Supplementary material for: The two kinases, AbrC1 and AbrC2, of the atypical two-component system AbrC are needed to regulate antibiotic production and differentiation in Streptomyces coelicolor
Source: Front Microbiol. 2015 May 12;6:450. doi: 10.3389/fmicb.2015.00450 (PMC4428217; doi:10.3389/fmicb.2015.00450)
Supplement: Supplementary file 3 [file Table3.DOCX]

***Table S3 - Oligonucleotides used in this work***

| **Name** | **Sequence 5’-3’** | **Use** |
| --- | --- | --- |
| SRG-001 | TTTTTTCATATGGAGGACAGGGTGCGGGTG | Forward oligonucleotide to amplify *abrC3* (NdeI site underlined). |
| SRG-002 | TTTTTTCTCGAGGGAATTGAGATAGGTCAGCACCGC | Reverse oligonucleotide to amplify *abrC3* (XhoI site underlined). |
| SRG-005 | GAGCGCGACCTGGCCGACGGGGCAC | Forward oligonucleotide for punctual mutation *abrC1*-H214A and *abrC2*-H270A. |
| SRG-006 | GTGCCCCGTCGGCCAGGTCGCGCTC | Reverse oligonucleotide for punctual mutation *abrC1*-H214A and *abrC2*-H270A. |
| SRG-022 | AGCGCGACGAGGAGAGGGACGGCACCGATGATTCCGGGGATCCGTCGACC | Forward oligonucleotide to obtain the mutagenic cassette for *abrC2*. |
| SRG-023 | GCACAGCCGTAGCGCGACGAGGAGAGGGAC | Forward oligonucleotide to elongate the mutagenic cassette for *abrC2*. |
| SRG-024 | CCCCCCGTCACGGCGCCGCTGTGCCGGTCATGTAGGCTGGAGCTGCTTC | Reverse oligonucleotide to obtain the mutagenic cassette for *abrC2* and *abrC1/C2*. |
| SRG-025 | ACGATGCCGTCCCCCCGTCACGGCGCCG | Reverse oligonucleotide to elongate the mutagenic cassette for *abrC2* and *abrC1/C2*. |
| SRG-026 | GTCGGCGTCTGCGGAGTGGGGTTTTCCCTATGTAGGCTGGAGCTGCTTC | Reverse oligonucleotide to obtain the mutagenic cassette for *abrC1*. |
| SRG-027 | CATGGAGCCCGTCGGCGTCTGCGGAGTGGG | Reverse oligonucleotide to elongate the mutagenic cassette for *abrC1*. |
| AY-008 | TGGCGCGGCGTCGCGCCGCACGGCACGCTGACGTGTATGATTCCGGGGATCCGTCGACC | Forward oligonucleotide to obtain the mutagenic cassette for *abrC1* and *abrC1/C2*. |
| AY-015 | CGGTCCACCTCATGGCGCGGCGTCGCGCCG | Forward oligonucleotide to elongate the mutagenic cassette for *abrC1* and *abrC1/C2*.. |
| AY-093 | TCGTGGTGGCG**G**C**C**GTACGGATGCC | Forward oligonucleotide for punctual mutation *abrC3*-D61A (bold). |
| AY-094 | GGCATCCGTAC**G**G**C**CGCCACCACGA | Reverse oligonucleotide for punctual mutation *abrC3*-D61A (bold). |
| AY-095 | TTTTTTCATATGGTGCACGGGCTGCTGG | Forward oligonucleotide to amplify *_c_abrC2* (NdeI site underlined). |
| AY-096 | TTTTTTCTCGAGGCCGGTGAGCGGCTCGTTCC | Reverse oligonucleotide to amplify *_c_abrC2* |
| AY-097 | TTTTTTCATATGGTGCGGGGGCTGCTGTC | Forward oligonucleotide to amplify *_c_abrC1.* NdeI site underlined). |
| AY-098 | TTTTTTCTCGAGCCCCCTGGGGCGCGAGCC | Reverse oligonucleotide to amplify *_c_abrC2* |
| SRG-041 | TTTTTTGAATTCGTGCGCCCCTCGCTCCCTGTC | Forward oligonucleotide to amplify *abrC1* promoter (EcoRI site underlined). |
| SRG-042 | TTTTTTCATATGACACGTCAGCGTGCCGTGCGG | Forward oligonucleotide to amplify *abrC1* promoter (NdeI site underlined).. |
| SRG-054 | CGTGGTGGCGGA**A**GTACGGATGC | Forward oligonucleotide for punctual mutation *abrC3*-D61E (bold). |
| SRG-055 | GCATCCGTAC**T**TCCGCCACCACG | Reverse oligonucleotide for punctual mutation *abrC3*-D61E (bold). |
| SRG-061 | TTTTTTCATATGGAGGACAGGGTGCGGGTGGTCATCGCCGAGG**CG**TCAGTGCTGC | Forward oligonucleotide for punctual mutation *abrC3*-D12A (NdeI site underlined). |
| HR-004 | CATATGGTGCGCCCCTCGCTCCCT | Forward oligonucleotide to amplify *abrC1* (NdeI site underlined). |
| EFE-002 | AGATCTCTACCCCCTGGGGCGCGAGC | Reverse oligonucleotide to amplify *abrC1* (BglII site underlined). |
| 4596-F | AAGACCATCACCGAGCTG | Forward oligonucleotide for qRT-PCR of *abrC3*. |
| 4596-R | GTAGCGCTCCTCCACGTA | Reverse oligonucleotide for qRT-PCR of *abrC3* |
| 4596-P | GTCGTGGTGGCGGACGTAC | Probe for qRT-PCR of *abrC3* |
| 4597-F | G CTGTGGTTCTGGGTGTT | Forward oligonucleotide for qRT-PCR of *abrC2*. |
| 4597-R | GATCTCGAAGGGGTTGTC | Reverse oligonucleotide for qRT-PCR of *abrC2* |
| 4597-P | AGGACGGGCTCCAGCTGTAC | Probe for qRT-PCR of *abrC2* |
| 4598-F | GGCACACCTGGAAAGAGA | Forward oligonucleotide for qRT-PCR of *abrC1*. |
| 4598-R | ATCACCGTGACCGTCAAT | Reverse oligonucleotide for qRT-PCR of *abrC1* |
| 4598-P | CGGCTTCACCTACGTGGTGA | Probe for qRT-PCR of *abrC1* |
| 4597-8-F | GGGTAGGGAAAACCCCACT | Forward oligonucleotide for qRT-PCR of intergenic region between *abrC1 and abrC2* . |
| 4597-8-R | CCGTAACCCTGTCCGTATTC | Reverse oligonucleotide for qRT-PCR of intergenic region between *abrC1 and abrC2* . |
| 4596-7-F | CGGGACTTCCCGTTCTCT | Forward oligonucleotide for qRT-PCR of intergenic region between *abrC2 and abrC3* . |
| 4596-7-R | CCTGTCCTCCACGATCCTC | Reverse oligonucleotide for qRT-PCR of intergenic region between *abrC2 and abrC3* . |
