# Supplementary figures and images for: The two kinases, AbrC1 and AbrC2, of the atypical two-component system AbrC are needed to regulate antibiotic production and differentiation in Streptomyces coelicolor
Source: Front Microbiol. 2015 May 12;6:450. doi: 10.3389/fmicb.2015.00450 (PMC4428217; doi:10.3389/fmicb.2015.00450)

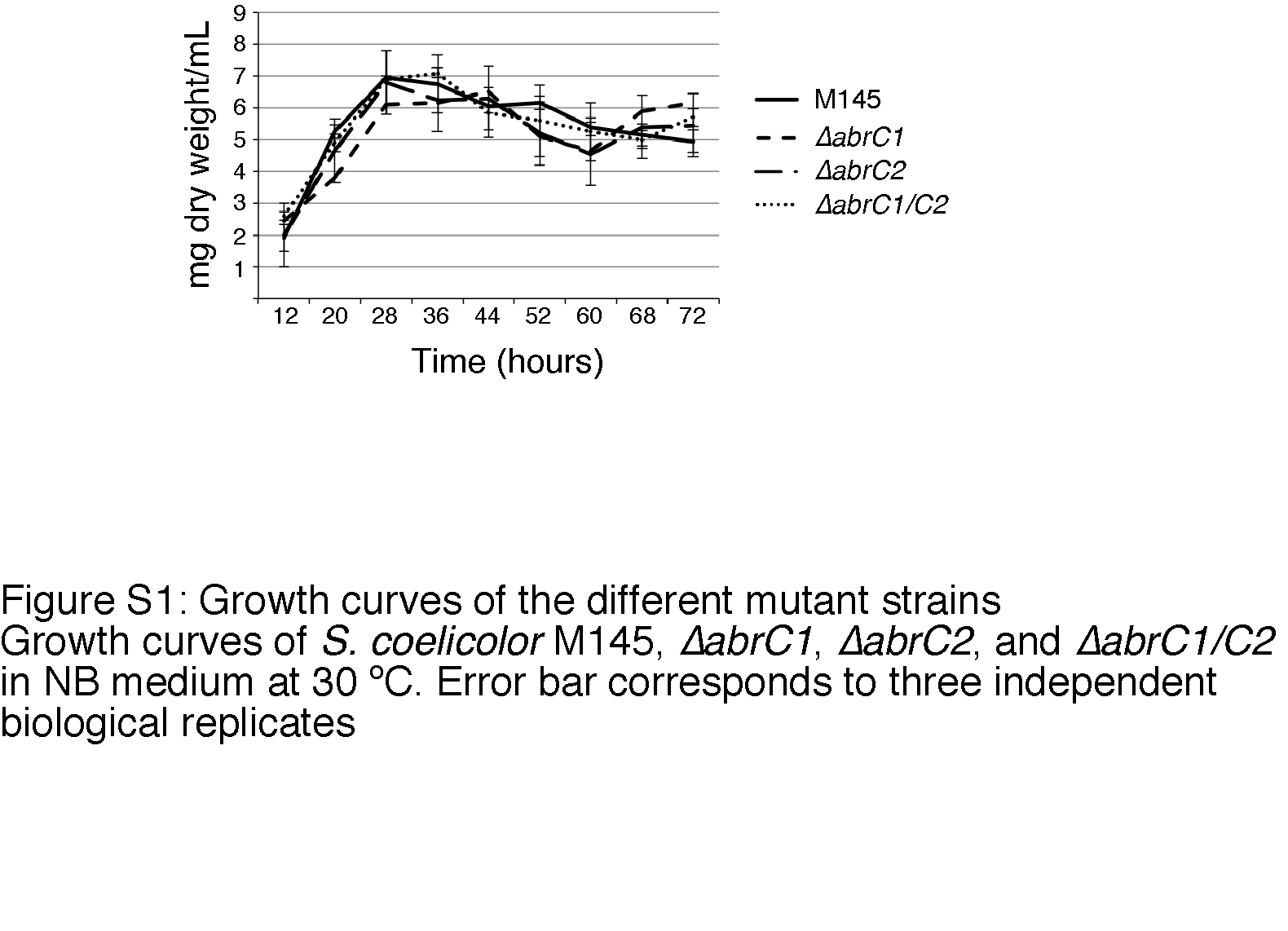

Supplement: Supplementary file 4 [file Image1.TIF]

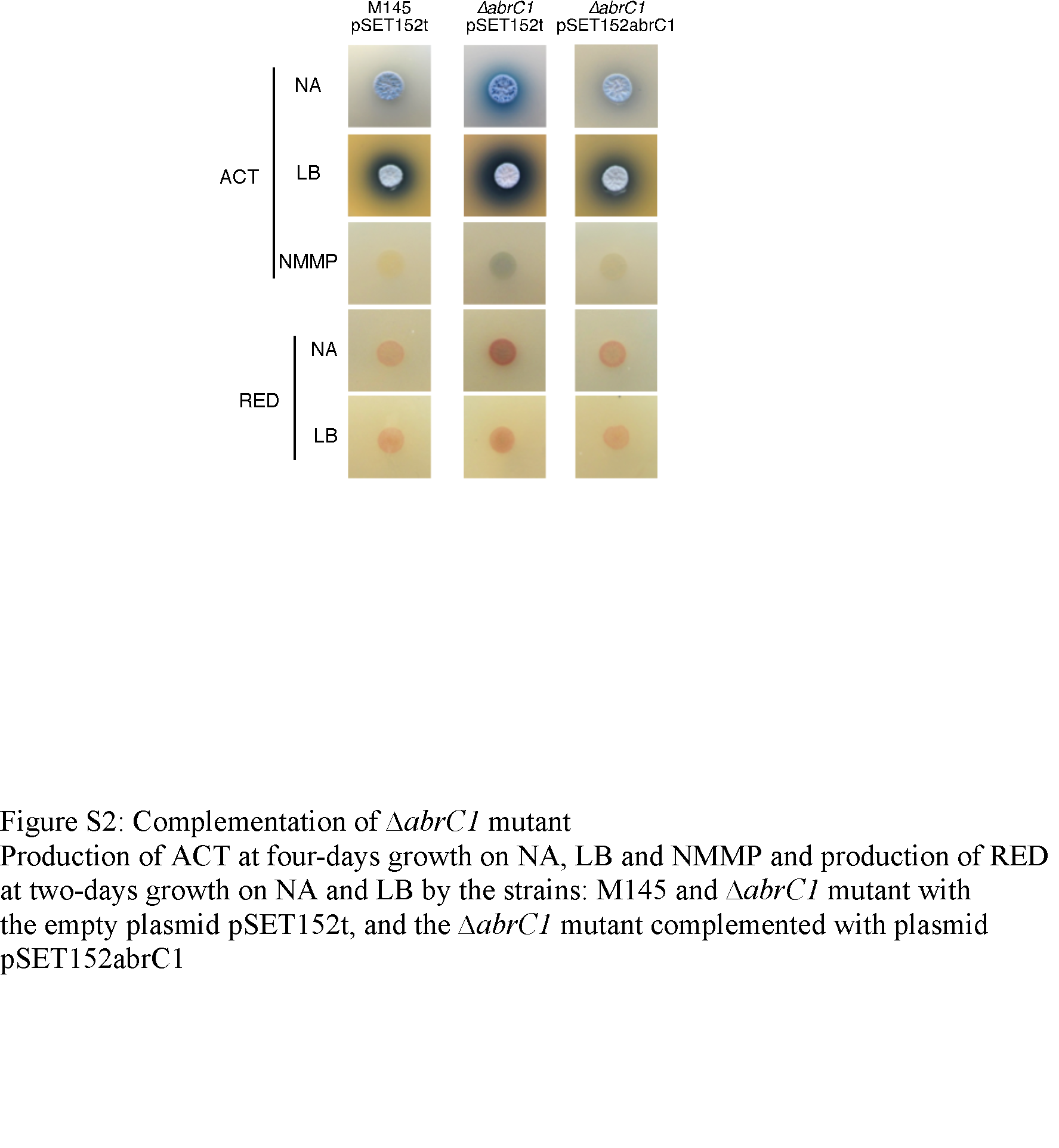

Supplement: Supplementary file 5 [file Image2.TIF]
